# A continuum of individual-level factors that influence modern contraceptive uptake and use: perspectives from community members and healthcare providers in Durban, South Africa

**DOI:** 10.1186/s40834-023-00247-7

**Published:** 2023-10-03

**Authors:** Yolandie Kriel, Cecilia Milford, Joanna Paula Cordero, Fatima Suleman, Petrus S. Steyn, Jennifer Ann Smit

**Affiliations:** 1 WWMRU (Wits MatCH Research Unit), Department of Obstetrics and Gynaecology, Faculty of Health Sciences, University of the Witwatersrand, Durban, South Africa; 2 School of Public Health and Nursing, College of Health Sciences, University of KwaZulu-Natal, Durban, South Africa; 3 UNDP-UNFPA-UNICEF-WHO-World Bank Special Programme of Research, Development and Research Training in Human Reproduction (HRP), Department of Sexual and Reproductive Health and Research, World Health Organization, Geneva, Switzerland; 4 Discipline of Pharmaceutical Science, College of Health Sciences, University of KwaZulu-Natal, Westville, South Africa

**Keywords:** Contraception, South Africa, Continuum of use, Individual-level factors, Family planning

## Abstract

**Background:**

South Africa faces numerous sexual and reproductive health challenges that can be mitigated with contraceptive use. Contraceptive use is defined and measured as use, non-use, or discontinued use. Research has shown that there are expanded definitions of use beyond these categories. Identifying such categories may assist in a better understanding of factors that influence contraceptive use.

**Setting and methodology:**

This qualitative study was conducted in the eThekwini Municipality in KwaZulu-Natal, South Africa. The aim was to explore the factors influencing the uptake and use of modern contraception. One hundred and twenty-seven participants were enrolled in this study. One hundred and three of those were community members, and twenty-five were healthcare providers. Focus group discussions and in-depth interviews were conducted to gather the data. Data analysis was facilitated using NVivo 10 software.

**Results:**

The data show that numerous factors influence contraceptive uptake and use. From these factors, a continuum of use that captures a variety of states of use emerged. Five different states of use were uncovered: no-use, vulnerable use, compelled use, conditional use, and autonomous use. The development of the model illustrates the complexity of contraceptive needs and that it extends beyond definitions found in policies and large-scale surveys. Expanding conceptions of use can aid in developing counselling and information support tools that can improve the uptake and continued use of modern contraception.

## Background

Unintended pregnancies remain a problem in Low and Middle-Income Countries (LMICs) where the unmet need for modern contraception is persistently high. An estimated 49% of all pregnancies in LMICs are unintended, and in 2020, 218 million women living in these regions had an unmet need for modern contraception [52]. Sub-Saharan Africa (SSA) has the highest rates of unintended pregnancy at 33.9%, with an unwanted pregnancy rate of 11.2% and a mistimed rate of 22.1% [7]. Unintended pregnancy is a high risk for abortions, many of which are unsafe and contribute to maternal deaths [7, 46, 52].

South Africa (SA) has progressive laws and policies that support the free provision of modern contraception to anyone over the age of 12, with a focus on improving the quality of care in sexual and reproductive health (SRH) services and the role that contraceptive use plays in the prevention of maternal mortality [34]. Yet numerous SRH challenges persist.

In SA, more than half of all pregnancies were reported as unintended, 20% were categorised as unwanted, and 35% as mistimed [38], which is concerning. The unmet need for contraception among unmarried young women is also high at 28% [38]. While the total fertility rate (TFR) in SA is the most advanced in SSA, there are significant differences between the various race groups in SA [37, 43]. Black South African women have a TFR of 2.7, while the TFR for White South African women is only 1.5 [38]. In addition, the modern contraceptive prevalence rate (mCPR) is only 48% for all women – significantly lower than in other middle-income-countries [34, 38]. The maternal mortality rate in SA has been rising over the past two decades, with the maternal mortality rate as reported in the SADHS 1998 being 150 deaths per 100 000 live births compared to the SADHS 2016 data that reported that figure has more than doubled to 536 deaths per 100 000. [16, 17, 38]. This difference was found to be statistically significant, illustrating the rise in maternal deaths. Although the HIV/AIDS pandemic and now the COVID-19 pandemic have had devastating effects on maternal mortality in SA, many of these deaths could have been prevented with modern contraception [16, 17, 56]. Finally, SA still has the largest HIV epidemic in the world. Young women between the ages of 15 and 24 who reside in the KwaZulu-Natal province, where this study was conducted, have the highest HIV incidence rates [11, 23, 55].

A factor to consider when examining contraceptive use is the preference for methods used in SA. There is a greater preference for short-acting hormonal contraceptives such as the Depo medroxyprogesterone acetate (DMPA) three-monthly injection and the two-monthly norethisterone enanthate (NET-EN) injections. DMPA is the most popular method at 18%, followed by the contraceptive pill at 8% [38]. The Implanon NXT Implant, introduced in 2016, saw good early uptake but then was followed by a period where removal rates were relatively high [9]. Hormonal contraceptives have known side effect profiles that can impact continued use if adequate counselling and information are not provided [14, 45]. This was evident with the Implanon NXT roll-out, where high rates of early removals were linked to side effects and users not being well informed about what they could expect [2, 9].

Factors that influence contraceptive use are not always harmful but can also be positive. The benefits of contraceptive use are far-reaching on macro- and micro-levels [18]. On the macro level, contraceptive use may benefit the environment, improve economies, decrease overpopulation, and alleviate poverty [18]. Unsafe abortions and maternal death would significantly decrease if all women in LMICs used effective contraception [52]. On an individual-micro level, reasons to use contraception include benefits to overall health, completion of schooling, improved economic situation, planned families and spacing of children [18, 52].

Despite the far-reaching benefits of using contraception, numerous reasons for non-use and discontinuation of contraception have been cited [14, 23, 39]. These include side effects, partner opposition, postpartum amenorrhea/breastfeeding, infertility, lack of access, and lack of knowledge [50]. Other reasons cited for discontinuing contraceptive use included costs, unavailability of methods, partner disproval, inconvenience, and infrequent sex [10]. In SA side effects are the leading cause for discontinuation at 28.4% [38]. Other factors include wanting to become pregnant (19.3%), wanting a more effective method (10.7%), becoming pregnant while using contraception (9.3%), and infrequent sex (8.3%) [38].

Lack of quality information is an often-cited reason for the non-use of contraception – especially among adults, adolescents, and men [12, 15, 39]. Poor counselling where contraceptive users are not properly informed about side effects can result in the discontinuation of contraceptive use [44].

Age is another important factor. Older women tend to have fewer unplanned pregnancies and use contraception more when compared to younger women [58]. Young women and adolescent girls have higher rates of unmet need which is mainly attributed to gender inequality and poor socio-economic situations [15].

Cost of obtaining contraception and accessing SRH services is another reason that limits the uptake of contraception. Costs, including transport, can be a significant barrier even in settings like SA where contraception is provided free of charge. Research has found that women with higher socio-economic status have higher rates of contraceptive use, while poorer women report lower use rates and therefore have an increased risk for unintended pregnancies [15, 59].

Side effects are often the key reason why women stop using a contraceptive method. Bleeding irregularities are the most commonly reported side effects which present as either amenorrhea, dysmenorrhea, or menorrhagia [14, 51]. Other side effects reported include weight gain, headaches, mood irregularities, and cardiovascular problems such as thrombosis [12, 14]. Side effects are also linked to numerous other aspects of contraceptive use such as finances, religion, relationship dynamics, cultural beliefs, and knowledge and counselling [14, 29].

As mentioned, SA, and specifically KwaZulu-Natal where this study was conducted, still has a increasing HIV incidence rate among young women (20–24 years, incidence rate 8.63 per 100 person-years) and adolescent girls (15–19 years, 7.79 per 100 person-years) [11]. HIV positive status has a significant influence on maternal mortality, as nearly 40% of maternal deaths are related to HIV/AIDS [16, 17]. Some of these deaths could have been averted with contraceptive use, as research has found that women who are HIV positive tend to have higher rates of unplanned pregnancies – indicating that they have a higher unmet need for contraception [28]. Recent initiation of antiretroviral treatment (ART) has also been associated with increased unmet need [49]. This could possibly be because healthcare providers are still uncertain about interactions between ART and some hormonal contraceptive methods [3, 20].

Contraceptive use is usually classified in the literature as either use, no-use or discontinuation ([8] {[14] #1). As with other health seeking behaviour, SRH services and contraceptive use is influenced by macro level factors (policy, healthcare system, and socio-cultural) or micro individual level factors [5]. An examination of the literature shows that little distinction is made between factors that influence non-use and those that influence discontinuation. There is a need to understand contraceptive use patterns outside the view of just use, no use, or discontinuation [50]. This is important to explore because contraceptive users and potential users may evaluate their need for contraception differently to the way from policies define their need [4]. Thus, an expanded view of contraceptive use can greatly assist with identifying gaps in patterns and dynamics of use. Exploring factors that threaten use is key to improving the uptake and continued use of modern contraception. The aim of this paper is to explore individual micro-level factors that influence contraceptive uptake and use at an individual level, and how these factors relate to each other on a continuum of use.

### Methodology

This study was conducted between 2015 and 2016 in the eThekwini Municipality, in the province of KwaZulu-Natal, South Africa. It formed part of a larger multi-country project between South Africa, Zambia, and Kenya. The project, known as the UPTAKE project, investigated the uptake and use of contraception to inform the development of an intervention to improve met need for contraception through a human rights and community approach using a theory of change [19]. The data presented here is only from the South African site.

### Recruitment

One hundred and twenty-seven female and male participants were recruited from two areas within the eThekwini Municipality. Area 1 was classified as an urban area, whereas area 2 was more peri-urban/rural. Purposive snowball sampling was used to find and recruit the participants. Project staff members entered both areas and communicated with key gatekeepers who assisted with recruitment of participants. Selection criteria for participants were determined by the type of FGD group. For the six community groups, their inclusion was determined by their area of residence (urban/peri-urban rural), and age (adolescents 15–19 years, young adult 20–34 years, and adult 35–49 years). For the three additional community groups, inclusion criteria included being not married/ no in-union; being married/in-union; and not have children. Participants did not need a prior history of being sexually active or using contraception to join the study. Health care participants were grouped according to their rank in their facilities.

### Participants

Fourteen focus group discussions (FGDs) and eight in-depth interviews (IDIs) were conducted in total. Focus groups allow for diverse opinions to be heard on a specific topic. The interaction between group members is as important as the questions answered as it allows for topics to be explored from a variety of angles [54]. In IDIs there is a detailed discussion between the interviewer and respondent. During IDIs, topics can be explored in depth through open questioning [54].

Community members who were both users and non-users of contraception, and healthcare providers participated in the FGDs. Key informants, who ranged from community key stakeholders to senior healthcare officials, participated in the IDIs. Table 1 shows the breakdown of the FGDs, and Table 2 shows the IDIs.

**Table 1:** FGD participants categories and numbers

FGDs conducted	No. of participants (n)
1. Females, urban, teenagers (aged 15–19 years)	9
2. Females, rural, teenagers (aged 15–19 years)	10
3. Females, urban, young adults (aged 20–34 years)	8
4. Females, rural, young adults (aged 20–34 years)	10
5. Females, urban, adults (aged 35–49 years)	8
6. Females, rural, adults (aged 35–49 years)	7
7. Males, teenagers (aged 15–19 years)	10
8. Males, young adults (aged 20–34 years)	8
9. Males, adults (aged 35–49 years)	7
10. Females who are unmarried/not in union, single (20–34 years)	8
11. Females who are married/in a relationship > 1-year (20–34 years)	10
12. Females with no children (who are not infertile) (18–49 years)	8
**Total community participants**	**103**
13. Health Care Providers (HCP) from local health facilities (including management, professional nurses): Group 1	8
14. HCP from local health facilities (including enrolled nurses, counsellors, and other operational staff): Group 2	8
**Total HCP participants**	**16**

**Table 2:** IDI participant descriptions

Key informants	No. of participants (n)
1. Educators	1
2. Community Care Givers	2
3. Traditional Healer	1
4. Programme Managers working in sexual and reproductive health (SRHR)	4
**Total key stakeholders**	**8**

### Ethics

This study (Project ID A65896) received World Health Organization (WHO) Ethics Review Committee (ERC) and Research Project Review Panel (RP2) approval. The University of the Witwatersrand Human Research Ethics Committee (Health- HREC, ref. no M1504101) provided local ethics review and approval. The University of KwaZulu-Natal's Biomedical Research Ethics Committee (BREC) provided reciprocity approval. The KwaZulu-Natal Provincial Department of Health granted permission to interview health care providers. All participants voluntarily signed an informed consent form, including permission to audio record the interview/group session. Parents or legal guardians granted consent for minors (those aged < 18 years) to participate. The minors provided assent. Participants were provided with $13.60 (at time of exchange rate) as reimbursement for their time and to cover their travel expenses. Food was provided during each FGD. 

### Data collection 

Study team members who were fluent in isiZulu and English were trained on the interview guides and conducted the FGDs and IDIs. The interviewers were matched according to age and gender with FGD and IDI participants to facilitate communication and data collection. The interviewers were all isiZulu and familiar with the research topic. They were extensively trained on interviewing and probing skills. The researchers who analysed the data consisted of a research psychologist and a research nurse, both whom have extensive experience within the field of sexual and reproductive health.

Audio recordings were made of each FGD and IDI. These were later transcribed and translated where necessary. The transcripts were reviewed and checked by a researcher for accuracy and any ambiguity was discussed and clarified with the interviewer.

### Theory and theoretical framework

Social constructionism was employed in this study through which to view the data. Social constructionism is interested in the collective construction of reality and the process of meaning making [13]. This stance allowed for various perspectives to be heard, as social constructionism argues that all views on a topic are valid [42]. Different actors may construct various realities on the same topic. This neutral type of stance was important since there was a variety of participants from various sectors of the community who participated. Social constructionism also allows for debates about certain truths surrounding sexual practices, including the use of contraception.

For the data presented in this paper, Andersen’s [5] health services utilisation behaviour framework was used. The use of healthcare services is complex and multifaceted. Macro factors are engaged in the process of accessing and using services that include policy, the environment, characteristics of the population, and the healthcare system [1]. This framework argues that for use of health services (in this case contraceptive service) to occur, predisposing characteristics, factors that enable or prevent use, and the need for care are required [5]. The Andersen model has four main domains – the environment, population characteristics, health behaviour, and outcomes. Individual characteristics play an important part in this model as it is the main unit of analysis. Predisposing characteristics, enabling resources, and need to use health services are employed in this framework to examine individual level characteristics that may influence the use of health services.

### Analysis

A team of coders were involved in the analysis of the data. A detailed account of the teamwork involved in this project can be found at Milford et al. [36]. A codebook was created after a subset of the transcripts were read by the researchers from the three countries. Another subset of transcripts was then double-coded, testing the master codebook for applicability, and to test for intercoder reliability. Differences in coding were discussed amongst the researchers until agreement was reached. In-depth coding was conducted using NVivo 10 (version 10, QSR International).

The initial round of coding was done using the constant comparative method, after which thematic content analysis was done to identify the larger themes [47]. After the initial round of coding was complete, advanced coding queries were run in NVivo 10 to uncover themes in the data. Matrix coding queries allowed us to explore the intersection of codes. Codes of influencing factors such as barriers and motivations to use contraception were iteratively coded. Patterns of use were elucidated in the data and placed within categories that include: no use, stopping, changing, and inconsistent use. From these categories a larger model was derived, as presented in the results section. Being cognizant that quantification of qualitative data can be misleading, each coding query was carefully read for relevance [48]. In instances where the text did not reflect the intersected codes, the text was un-coded as the specific code (s).

The overall results of the project were shared with the community during a community feedback session. The agreement from the community with the findings were determined with the use of structured assessment guide and an active feedback session with the community members and key stakeholders. There was a high degree of agreement with the study findings from the community and healthcare providers’ perspectives, which added validity to the findings.

### Demographic results

One hundred and three community participants were involved with this study, consisting of 25 males and 78 females. The mean age for the males was 23.8 years and the females 26.4 years. Unplanned pregnancy was high in this study population, as 73.7% (*n* = 45) of the female and 87% (*n* = 7) of the male participants who reported a pregnancy, classified them as unplanned. Contraceptive use was high with 83.5% of participants reporting some form of modern contraceptive use, either in the past or currently. Male condoms were the most commonly used method (75%, *n* = 65), followed by the depo medroxyprogesterone acetate (DMPA) injection (36%, *n* = 31).

### Thematic results

**Fig. 1:**
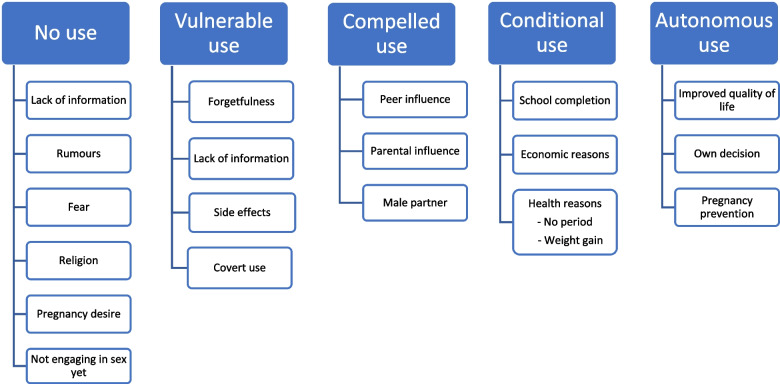
Model showing the components of the continuum of contraceptive use

A model (Fig. 1) was created that aided with contextualising the findings. The boxes capture the overarching themes, while the circles show the thematic codes that were used. Five components were established from the data: No use, vulnerable use, compelled use, conditional use, and autonomous use. The components of the model demonstrate how barriers and motivations to use contraception can move along a continuum and that they can have different outcomes in various contexts. An overlapping of barriers was noted as shown in Fig. 1 below.

### No use

The most significant barriers reported as no-use barriers were related to the healthcare system. These factors that broadly include access and quality of care factors are explored in detail elsewhere, see Kriel et al. [32], and Kriel et al. [33]. Individual-level factors included lack of information, rumours, fer, religious beliefs, pregnancy desire, and not having engaged in sex yet.

Lack of information was a widely cited reason why people do not use contraception. One participant explained that they are often not aware of the available methods:

*You would say or say hay I do not know what is there, and they would say oh you don’t know the thing you came to inject with you see? They do not explain to you everything and give you information and say there is this and that and that and that and that.*

*[Females without children, FGD, P3]*

An adolescent participant explained how unintended pregnancies can happen because young people do have adequate information about contraception:

*But then we do not sleep [have sex] because we want kids, mistakes happen, (Group laughs). You see, you [have] sex thinking that you are just stealing a bit. It would just happen, maybe by mistake you get pregnant, but that you won’t be aiming [for] that. You are still new in this thing, so you would [not] know that you have to go to the clinic and get an injection. You will see when it is late maybe after you got pregnant that ‘hey I should have gone to the clinic’.*

*[Rural Adolescent Females, FGD, P1]*

One participant’s explanation on forgetfulness, and rumours and misinformation about side effects demonstrated their decision to not use contraception:

*Yes me, as I said I used to forget pills, and the three years injection (Implant) I usually hear that it kills, this and that the Depo injection makes you wet. So I’m not using anything, I hear a lot of things so.*

*[Rural Young Adult Females, FGD, P9]*

Fear of different methods was a reason for non-use. Another participant reported how her fear of female condoms prevented her from using them:

*It is just that the female one (condom) is not common. I don’t know if it is not common because it is scary. I become scared of the way it is presented, the way you use it […] I saw it, you become afraid to give it to a man.*

*[Rural Adult Females, FGD, P4]*

Fear of being injected was cited as another reason to not use injectable contraceptives:

*I have never tried it (injectable contraception). My problem now is that I am a coward (all laughing). I have never tried. I use condoms because I’m scared of being injected.*

*[Married Females, FGD, P5]*

Religious beliefs can also prevent contraceptive use as one young adult participant explained:

*I say it is there in religion because, especially the born again [Christians], isn’t that a person is not allowed to have sex with a person if they are not married. But you find […] there is a person she has sex with. Now she can’t go do family planning because she will be scared that in the clinic people that know her, will see her that she is born again but you do family planning. You find that she in that way fall pregnant.*

*[Rural Young Adult Females, FGD7]*

Another important reason to not use contraception was the desire to have a baby as this participant pointed out:

*There are those who say, ‘I don’t see the need and will not inject [with injectable contraception] because I do not have a baby yet’, which means [there is] a decreased chance of having a baby, let me have a child first.*

*[Rural Young Adult Females, FGD, P9]*

Some young adolescent participants reported that they had not begun using contraception because they had not yet engaged in sex.

***F: Why you are not preventing pregnancy? Not using contraception or not using family planning, can you tell me?***

*P10: Because I have not started having sex as yet.*

*P6: I have not started having sex as yet too.*

*[Rural Adolescent Females, FGD]*

However, other adolescents pointed out that it is important to use contraception even if they are not having sex at the time:

*Can I please say this, if you are on family planning that does not means you have to do family planning because you have started having sex. You have to do family planning because you will say ‘I don’t do family planning, not do it today’, [but then] go to a boy, after that the boy will break you and get [you] pregnant.*

*[Rural Adolescent Females, FGD, P7]*

### Vulnerable use

Vulnerable use is use that is threatened or disrupted in some way. Reports of discontinuing (stopping), changing, interrupting, and covert use of methods showed how vulnerable use can influence contraceptive use patterns. Vulnerable use patterns were most commonly reported in the data.

Forgetfulness was a key factor that resulted in inconsistent use and was mainly discussed with regards to the oral contraceptive pill, but also included discussions about missed visits for either re-injection or follow-up for the Implant.

One female participant explained how gendered roles and tasks within the household can result in forgetting to take contraception:

*I am at home I have to take a pill [contraceptive pill] maybe there are lot of things in my head I must bath the children, wash the dishes, I must cook you see, there is too much, I will forget that I have to take pills.*

*[Rural Young Adult Females, FGD]*

Another female participant explained that if a person forgets to take their oral contraceptive pill at the correct time, they can fall pregnant easily:

*You have to have an exact time. If you drink them [referring to the oral pill] at 8, it’s always 8 for all on that time. Because […] if that time comes and you forget the time, you can easily get pregnant.*

*[Women without children, FGD P3]*

Participants also pointed out that forgetfulness can interfere with the effectiveness of injectable contraception. One participant described how people can fall pregnant while on injectable contraception because they forgot their re-injection date:

*[H]ow do you get pregnant while on contraceptives? It is you who miss your injection dates, or you fail to wait for the time.*

*[Rural, Adult Female FGD, P5]*

Another participant described how she uses her phone to remind her of her re-injection date in order to avoid having another unintended pregnancy:

*I make sure that I set alarm on my phone so that on the 4*^*th*^*I go to the clinic because I don’t want to repeat the mistake of falling pregnant again […].*

*[Rural Young Adult Females, FGD, P4]*

There were also reports that forgetfulness can play a key role in returning to the clinics for removal and replacement of the Implant:

*With 3 years [Implant] you forget. You can insert it today I am sure that you don’t count that okay it’s 2015, 2016 and 2017 I have to go back. You forgot long time ago, and you have missed your date.*

*[Urban Young Adult Females, FGD, P1]*

Lack of information could also lead to interrupted use as one participant explained:

*It means that problem that we are facing is lack of knowledge. (Mhh, exactly, yes all of them agreeing with her). It is the thing that we are facing because we listen to half and forget. If you have [a] running tummy and [you are] on contraceptives... [the] pills or injection gets interrupted in the system. That means people needs to be educated and know that other things [can] prevent [contraception from working]. And know that even what you are using is not 100% safe. So you have to do 1, 2 and 3 so that you are in the right situation.*

*[Married/in-union women, FGD, P6]*

Side effects were the most discussed reason for discontinued use. One female participant explained how menorrhagia influenced her decision to stop using contraception:

*I started with Depo. I injected with it, but I still continued bleeding because when the [contraceptive] pills started to make me sick I was bleeding and I was taking them every day. I'd bleed till the next collection date then go to the clinic so the doctor then said I must stop using them.*

*[Married Females, FGD, P4]*

Amenorrhoea was another side effect that resulted in discontinued use of contraception. This side effect was often associated with the belief that the womb is ‘blocked’, dirty or being damaged:

*Okay I was on the 2 months injection [referring to Nur-Isterate Injectable]. It was locking my periods […] I did not like that [..]. So, I decided to leave it because of [not having] periods. I think it is something that should come out sometimes. So yes, I then left it [Nur-Isterate injectable] because of that and I’m still not using it now.*

*[Women without children, FGD P4]*

One participant believed that amenorrhoea due to contraceptive use could lead to infections, and therefore it was important to discontinue contraceptive use:

*It ends up not being right. In others it damages the womb […] it is this blood that was supposed to…. this is the blood that is making you sick that is causing an infection […] that’s why you have to take it [referring to the Implanon NXT Implant] out when it supposed to come out. It’s the blood that has been stored all these years and it was not coming out.*

*[Women without children, FGD P3]*

Another associated amenorrhoea with being ‘dirty’:

*[S]he then stopped [her contraception], then she got her periods. [T]hose who are doing Life Science [referring to a school subject] they know that if you are a female there has to be blood coming out of your body, that is dirty, there has to be dirt that is coming out of the body.*

*[Rural Adolescent Females, FGD, P6]*

Weight gain was another important side effect, as one participant noted:

*I was injecting with Depo 3 months I stopped using it [contraception] because it was making me very fat. I moved from size 30 to size 34 I stopped […].*

*[Married Females, FGD, P7]*

There were numerous reports about changing contraceptive methods. These were mostly associated with side effects, as one participant explained:

*I left it [2-monthly injectable] because I heard that there was the 3-years [Implant]. I inserted the Implant, but it has lots of side effects. I’m even from the doctor because of the side effect. I just had to change it. The doctor said I must go back to 2-months I must change it.*

*[Females without children, FGD, P3]*

Covert use discussions were also raised under this theme as discovery of use could result in discontinuation. One participant described how she has to hide her family planning card from her male partner to continue using her contraception:

*USFG_C007: I go for injection come back and hide my card, on my date I go and come back there at the clinic, I don’t tell him.*

*USFG_C008: Because he wants a child.*

***Facilitator: The reason you hide the card is because he wants a child?***

*USFG_C007: Yes. I am not ready for a child.*

*[Females without children, FGD]*

### Compelled use

Compelled use reasons included discussions about external influences that could be the reason for use or no use. Examples of these included peer pressure, male partner influence, and parents insisting that their children use contraception. A notable factor in compelled use is that once the external force is removed contraceptive use will most likely stop.

One female participant from the married/in-union group explained how adolescents influence each other, and how males can pressure their female partners to not use contraception– and in particular to not use condoms. She also highlights how insufficient information about various contraceptive methods can result in unplanned pregnancies:

*A child [referring to an adolescent girl] is going to use a condom [but an adolescent] boy will convince her not to use it when they have sex. [She will then] get pregnant. If she knew about other methods she was going to use them because they fall pregnant at the age of 11 [and] 12.*

*[Married/in-union women, FGD, P4]*

Male participants in particular noted how peer pressure can influence contraceptive use. One adolescent male participant explains how friends and alcohol use can negatively influence contraceptive use (especially condoms):

*The thing that makes us not to go, as youth, dislike using these prevention methods it is the influence that we get from other people that we befriend, our peers who are- who don’t use these things. We as youth like to say that we have bad luck you see, but we put money together to buy alcohol and when we are drunk and we have sex without using prevention methods because we are influenced by this alcohol that we have been drinking.*

*[Adolescent Males, FGD, P4]*

One female participant, a parent, explained how she had taken her daughter to the clinic to get contraception. Once the mother’s influence was removed the daughter stopped using the method:

*I am the one who once went with my child. I just thought let me speak to the clinic sister because I am a mother. I went with her, and the clinic sister said it is better that she takes a decision on her own. I didn’t know too well, but I knew that I wanted her to use these pregnancy prevention methods. I initiated that she uses the injection, but what I have noticed is that she has stopped.*

*[Rural Adult Females, FGD, P4]*

### Conditional use

The participants described situations where contraceptive use was conditional and where a specific goal was the motivation to use contraception. These motivations included factors such as completion of school, economic factors, pregnancy prevention, and health related motivations such as not having a period or gaining weight.

An adolescent participant pointed out that using contraception was important so that they can complete school:

*In my view contraceptives are right, [especially if] you are still a student. If you are having sex, you must not get a child but continue with school.*

*[Rural Adolescent Females, FGD, P5]*

Male participants also cited the rationale for pregnancy prevention, including economic factors that influenced contraceptive use:

*My partner and I, we use condoms, when I am with her. Because the other thing I am trying to prevent is the issue of expenses, and she also understands that […] because life is not yet the way I want it. I am trying to run away from having children all over.*

*[Adult Males, FGD, P7]*

Another often reported reason to use contraception for purposes other than pregnancy prevention was to not have a period. A healthcare provider participant explained:

*Some they like family planning because some injections stop their periods, so any reason mainly she will not receive her periods.*

*[HCP, Group 2, P5]*

While gaining weight was a problem for some people, others found this to be a reason to use contraception, as a healthcare provider describes:

*There are people who like gaining weight. Some said ‘I heard that it makes people gain weight sister. I do not like this body I have it does not give me pleasure. I am asking for the prevention methods so that I will gain weight.*

*[HCP, Group 2, P6]*

### Autonomous use

Ideal use situations were discussed as motivations to use contraception and can be defined as autonomous use. These accounts captured situations where participants used contraception without any significant obstacles that could prevent or interrupt use. Pregnancy prevention, own decision making, and improving the quality of life were cited as examples of autonomous use.

Making decisions about contraception, own decision making, was raised as being important for a woman to use contraception autonomously:

*Really family planning is up to a woman, to think for herself. Males never think the way you think because at the end it us that feel more pain than males.*

*[Married Females, FGD, P2]*

One female participant explained how contraception helped her after she had had her first baby – highlighting how her decision making about pregnancy prevention was important to improve her quality of life:

*I think that family planning is the right thing especially nowadays because things are expensive. It helped me because I had a baby while I was still young. I went off family planning and got another baby after 10 years.*

*[Urban Adult Females, FGD, P6]*

Another female participant described how using contraception was important to increase the quality of her life:

*I like it [contraception] because if you’re using it sometimes you can be free that no I cannot get pregnant anyway, now I can continue making my dreams things I like to do [come true].*

*[Urban Young Adult Females, FGD, P4]*

## Discussion

Numerous factors that influence the uptake and continued use of contraception were identified in this project. However, for contraceptive use to be actualised, a need to utilise it must be present. This argument is according to the healthcare utilisation behavioural model that was used to analyse the data in this study [5]. This model demonstrates the complexity of healthcare utilisation, and in this case contraceptive use. The continuum of use is defined as the various potential stages of use that an individual can be classified in. A range of influencing factors determine which stage an individual may be in. This model builds on the individual characteristics component of the Andersen model [5, 6] by engaging the predisposing, enabling, and need components in the use of SRH health care services. Individual level characteristics is a key determinant of how and when individuals use modern contraceptive methods. Each component of the continuum is influenced by the degree of use or non-use, and does not necessarily present a linear flow. An individual can go from the autonomous use stage to non-use depending on the predisposing, enabling, or need factors that influence their use at that time. It is important to recognise that use varies over time, which is essential as contraceptive use occurs over numerous decades of a woman’s life. Exploring how the various factors that influence use fit along a continuum allows for a more robust understanding of the concept of use.

In the literature, contraceptive use is usually defined and evaluated as either use, no use, or discontinuation [8, 14]. Baumgartner et al. [8] argue that discontinuation can be classified as either intentional, due to side-effects or wanting to fall pregnant, or unintentional through missed appointments. While large-scale surveys can identify various factors that influence use, there is a need for qualitative inquiry to explore and contextualise how factors influence use [50].

In this study various factors that influence contraceptive use were reported. These factors were grouped thematically and a simple model demonstrating the various states of use emerged. This model captured five potential states of use namely: non-use, vulnerable use, compelled use, conditional use, and autonomous use. It is possible that more than these five states exist. There was considerable overlap of influencing factors between the states of use, illustrating the challenge in distinguishing whether factors result in no use, discontinued use, or interrupted use.

The first use state was *no-use* of contraception. According to the data, need may or may not be present or acknowledged. Key themes raised under no use included fear, lack of information, rumours, fear, religion, pregnancy desire, and not having engaged in sex yet. Lack of quality information was a widely reported problem that influenced numerous other states. Numerous studies have found that although people know about various contraceptive methods, such knowledge may not result in contraceptive use, the quality and type of information supplied seems to be important [24, 26].

Fear was another reported barrier that participants associated with non-use. Fear was mainly associated with the type of contraceptive method and its associated side effects. Participants reported that being scared prevented them from using contraceptive methods. The literature reports that fear can either be due to personal experience or the experience of others [50]. For non-users, reports of negative experiences by others can play an important part in not using contraception and increasing their risk for unplanned pregnancies [45].

The desire to have a baby was also included under the no-use category. When examining contraceptive use it is important to bear in mind that for many women and couples, pregnancy is highly desirable. Provision should be made to understand this motivation as it illustrates the continuum along which contraceptive use can move. Furthermore, being cognisant of this motivation during counselling can provide women and couples with the information they need to adequately plan their future family and how to use contraception successfully post-pregnancy.

The *vulnerable use* state contained reports of instances when women recognised the need to use contraception but faced significant obstacles that led to changing methods, discontinued and inconsistent use, and covert use. The vulnerable state was also the use state with the most reported factors influencing contraceptive uptake and utilisation, indicating that many women in this setting face significant obstacles to continual use of contraception.

Side effects were the most widely reported reason for either not using, stopping or changing a method. Participants explained that side effects often became intolerable and interfered with their daily lives, resulting in them discontinuing their contraceptive method, a finding supported by the literature [45]. The most commonly reported side effects in this study were bleeding irregularities and weight gain.

According to the literature, bleeding irregularities are the most common side effect related reason to discontinuing contraception [14, 38, 45]. Bleeding side effects can either be classified as menorrhagia, irregular bleeding, or amenorrhoea [25]. Concerns about physical health due to menorrhagia and its associated inconvenience resulted in the discontinuation of contraceptive use according to study participants. Amenorrhoea resulted in discontinued or interrupted use as women reported that they wanted to see their menses for fear of a ‘build-up of blood’.

The influence of these side effects extended beyond physical discomfort and can result in economic and interpersonal challenges [35]. They also illustrate how personal beliefs and inaccurate information can lead to misconceptions about side effects. Economically women who have menorrhagia or irregular bleeding will have to purchase additional sanitary wear. Bleeding side effects can also influence relationships as heavy, prolonged or irregular bleeding can impact sexual activity within relationships [27].

Weight gain was a particularly important problem for women in this setting, possibly due to its financial impact as women may have to purchase new clothes. Results from this study found that significant weight gain can lead to changing of methods and discontinued use of contraception. Weight gain is also reported in the literature as an obstacle to continued method use [14].

That side effects were so widely reported is related to the fact that short-acting hormonal contraceptives remain the most used contraceptives in SA [15, 38]. These contraceptive methods are known for their side effect profiles and highlight the need for a shift to longer-acting contraceptives such as the intrauterine device (IUD) [45]. People also need better counselling and information about what side effects to expect and the actions they can take to mitigate or decrease the side effects [22].

Another pattern of contraceptive use that was discussed under vulnerable use is covert use. Covert use has been reported in this setting and is linked to discontinuing contraceptive use if the male partner discovers such use [31]. Female participants in this study reported that using contraception covertly was a way to protect themselves from falling pregnant despite their male partners insisting on having more children. Covert use is also associated with decision-making. Although joint-decision making is the most common type of decision-making, the literature shows that female decision making is associated with higher rates of contraceptive uptake and use [21, 53]. Although covert use can facilitate female decision-making and contraceptive use it does pose a strong element of risk to continued use [31, 41].

*Compelled use* contained descriptions where an external force influenced the use of contraception. Individuals did not necessarily recognise the need to use contraception and once the external influence was removed, contraceptive use ceased.

One example of such compelled use was the influence of peers. Participants reported that peer influence can result in the non-use of contraception, especially where alcohol intake was involved. Adolescents in particular reported that their friends played a key role in them not using contraception. The literature shows that increased alcohol intake amongst adolescents lead to impromptu sexual encounters with decreased contraceptive use [57].

Parents play a particularly important role in the uptake and use of contraception. However, in some instances their influence can result in compelled use, as the parents may perceive the need for their adolescent children to use contraception rather than the adolescents themselves. Such compelled use was described by a mother who felt it was necessary for her daughter to use contraception, yet the daughter herself did not see the need to use it independently. This supports Andersen’s [5] assertion that need must be perceived by the individual for healthcare use to take place. If young adults and adolescents do not perceive the need to use contraception, they will have an increased risk of unintended pregnancies.

*Conditional use* involved discussions where people felt that they needed to use contraception out of necessity and often with a degree of reluctance. Use, and the need to use contraception, was usually linked to an external goal that they wished to achieve.

One of the key goal-linked motivations to use contraception was to complete school or some education. Higher levels and completion of education have been linked to increased contraceptive use [24]. Furthermore, adolescents who are not in school are twice as likely to not use contraception and have an unplanned pregnancy [30].

Another goal-motivating factor was the economic benefits of contraceptive use. Male participants reported that they supported the use of contraception to prevent additional expenses due to unplanned pregnancies. The literature also supports the finding that male partners are more likely to encourage contraceptive use due to financial reasons [31, 40].

The *autonomous use state* is ideal use, where the user is motivated and supported to use contraception consistently and successfully. Here the user fully recognises the need to use contraception and has the means and ability to overcome any obstacles that might inhibit use, as explained in the health utilisation belief model [5].

A key motivating factor to use contraception for pregnancy prevention was the improvement of a person’s quality of life. Participants in this study reported that preventing unplanned pregnancies and spacing their children had a positive effect on their lives and served as motivation to use contraception. Such general positive influence on quality of life as a motivation to use contraception is also reported in the literature [18, 52].

Own decision-making was also linked to autonomous use by the participants. Female participants encouraged women to make their own decisions to use family planning, as the consequences of unplanned pregnancies usually fall on women. This view, that women should have agency in decision-making and seek opportunities to use contraception is also supported by the literature [35].

Some limitations in this study should be noted. The findings are reported from a limited study population based in the eThekwini Municipality. Cultural beliefs are varied across South Africa and therefore the findings may not be applicable in other settings. The model that was derived from the data requires further testing and may not capture all states of use. That said, it does offer insight into the complexity of contraceptive use. Using the health utilisation behavioural model [5] is a useful start in understanding contraceptive utilisation. However, contraceptive use poses challenges for the model, as need for contraception differs significantly from disease-related need, and the benefit of contraceptive use extends beyond individuals [18]. Need for contraception ranges from real need to more abstract degrees of need. In some instances, people may not realise their need for contraception, as the case of the adolescents demonstrated. In other cases, need may be very clear where people are motivated and able to overcome a range of obstacles and barriers to use contraception. Evaluated need may also not be perceived as need by users, especially in the cases where women are not in relationships or have infrequent sex, but still fall under the definition of having an unmet need [50]. Such discrepancies between perceived need and evaluated need may result in the misclassification of a state of use [4].

## Conclusion

This study demonstrated that there are various possible states of contraceptive use that can be viewed along a continuum. The detailed exploration of factors that influence contraceptive use or non-use provides a better understanding of risk for unintended pregnancies. Contraceptive use depends on perceived need by the user and can be understood on a continuum model that ranges from no use to autonomous use. The use of a continuum facilitates the expansion of a narrow view of use or no-use definitions, which can assist with identifying women who are at risk of unintended pregnancies. Vulnerable use was the most frequently discussed state, with the most influencing factors, in this study, with side effects playing a significant role in discontinuation of use. However, most women will most likely fall under the conditional use state since it captures a broader definition of use. This model has the potential to shift policy and research focus from initiation to also include long-term continuation of contraception. It does this by highlighting the possibility of various possible states of use and how the state of use may fluctuate over time. When individuals are initiated on contraception, the long-term use should also be kept in mind. Further research is needed to explore these expanded definitions and their applicability to users.

## Data Availability

The datasets generated and analysed during the current study are not publicly available due to the sensitive nature of the data. For these qualitative data there is a high risk of compromising participant and health care system confidentiality but are available from the corresponding author on reasonable request.

## Abbreviations

DMPA: Depot medroxyprogesterone acetate
FGD: Focus group discussion
FP: Family planning
HCP: Health care providers
KI: Key informant
SA: South Africa
SRH: Sexual and reproductive health
